# Alzheimer’s Disease: Current Perspectives and Advances in Physiological Modeling

**DOI:** 10.3390/bioengineering8120211

**Published:** 2021-12-12

**Authors:** E. Josephine Boder, Ipsita A. Banerjee

**Affiliations:** Department of Chemistry, Fordham University, 441 E. Fordham Road, Bronx, NY 10458, USA; eboder@fordham.edu

**Keywords:** Alzheimer’s disease, biomimetics, neurodegenerative diseases, microphysiological systems

## Abstract

Though Alzheimer’s disease (AD) is the most common cause of dementia, complete disease-modifying treatments are yet to be fully attained. Until recently, transgenic mice constituted most in vitro model systems of AD used for preclinical drug screening; however, these models have so far failed to adequately replicate the disease’s pathophysiology. However, the generation of humanized APOE4 mouse models has led to key discoveries. Recent advances in stem cell differentiation techniques and the development of induced pluripotent stem cells (iPSCs) have facilitated the development of novel in vitro devices. These “microphysiological” systems—in vitro human cell culture systems designed to replicate in vivo physiology—employ varying levels of biomimicry and engineering control. Spheroid-based organoids, 3D cell culture systems, and microfluidic devices or a combination of these have the potential to replicate AD pathophysiology and pathogenesis in vitro and thus serve as both tools for testing therapeutics and models for experimental manipulation.

## 1. Introduction

**Figure 1:**
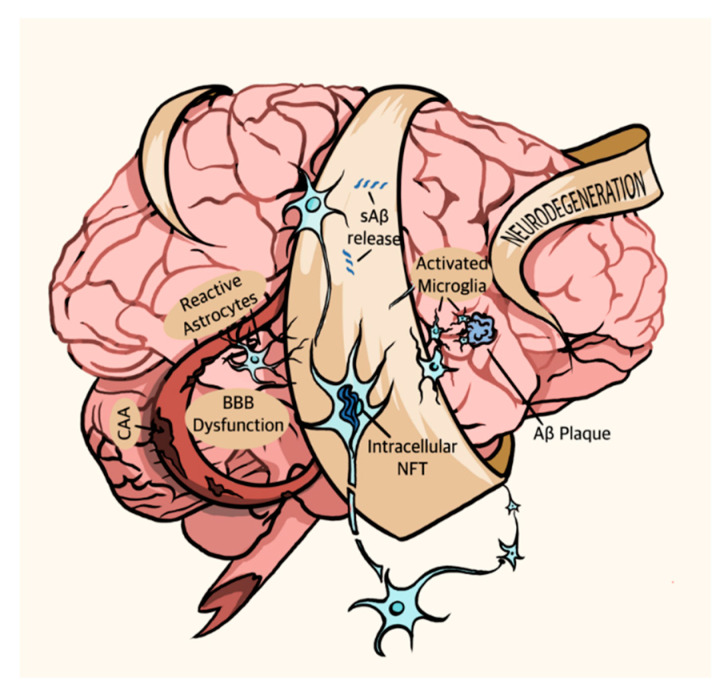
The AD brain. Pathophysiological symptoms of AD include soluble Aβ (sAβ) release, Aβ plaque deposition, activated microglia, intracellular NFT, BBB dysfunction, cerebral amyloid angiopathy (CAA), reactive astrocytes, and neurodegeneration.

Since its first description in 1906, both the scientific community and the general populace have become intimately familiar with the debilitating neurodegenerative disorder known as Alzheimer’s disease (AD). Characteristics of the disease include the formation of extracellular amyloid plaques composed of amyloid-β (Aβ) peptides and intracellular neurofibrillary tangles (NFT) composed of hyperphosphorylated tau (p-tau) protein in the brain [1]. These pathophysiological symptoms typically coincide with a slow progression of crippling clinical symptoms such as emotional disturbances, impaired cognition, ataxia, and eventually death (Figure 1) [2]. To date, most AD drugs offer treatments, usually in the form of cholinesterase inhibitors and NMDA receptor antagonists [3,4,5], depending upon the stage of diagnosis. These treatments may aid memory and slow disease progression but do not substantially modify the course of the disease. Clinical trials have indicated the efficacy of BACE1 (beta secretase) inhibitors in lowering amyloid β levels in the brain, however, the long-term effects of BACE1 inhibition on physiological well-being and in improving cognitive function in AD patients have not yet been fully understood [6]. Recently, however, Aduhelm, the anti-Aβ antibody aducanumab, became the first drug that can potentially remove amyloid plaques that build up in the brain and it was approved by the FDA; however, despite its demonstrated ability to reduce Aβ plaques in clinical trials, clinical evidence indicative of its ability to affect cognitive decline even in early-stage patients is inconclusive at best [7,8,9,10]. More recently FDA has approved Phase I clinical trials for nasal vaccines to prevent and slow the progression of the disease. The vaccine contains the immunomodulatory protein protollin that activates the immune system to induce clearance of β-amyloid plaques [11]. Unfortunately, many therapeutics consistently fail when they go to clinical trials despite early successes in AD models [12], indicating a need for both improved understanding of the processes underlying AD and improved experimental models of the disease. The recent advent of induced pluripotent stem cells and improved in vitro cell culture techniques sparked the creation of a vast array of microphysiological AD models, many of which show immense promise as both tools for testing therapeutics and models for experimental manipulation. 

### 1.1. Alzheimer’s Disease Pathophysiology

#### 1.1.1. Amyloid Beta Peptides (Aβ) 

The two major proteinaceous hallmarks of AD, Aβ, and p-τau, garner significant attention from researchers. The biochemical pathways by which they arise are well defined. Both proteins occur naturally in the mature CNS; in fact, amyloid plaques and NFT occur naturally (even if in smaller quantities) in the brains of aging adults [13]. 

Accordingly, the healthy CNS continually produces and clears Aβ [14], as do cultured cells [15], though the peptide’s physiological role is still subject to speculation. Current hypotheses hold that Aβ deposition occurs due to either an increase in overall Aβ production or an increase in the ratio of the more toxic Aβ42 isoform to the less toxic Aβ40 isoform [16]. The latter hypothesis rose to prominence more recently than the first and enjoys a growing body of supporting evidence, for example, amyloid plaques in deceased AD patient brains typically mostly consist of Aβ42 and not Aβ40, despite a relatively higher amount of soluble Aβ40 in their brain tissue [17].

#### 1.1.2. Tau

Tau is a cytoskeletal protein that, unlike Aβ, has a clear physiological role as a stabilizer of microtubules [18]. The neurofibrillary tangles that characterize Alzheimer’s disease (as well as related tauopathies) occur when Tau becomes hyperphosphorylated, forming p-τau. NFTs form within neurons, leading to their death—a likely reason why NFT pathology correlates with dementia severity [19]. P-τau also forms threadlike structures called neuropil threads when it accumulates in dendrites; these threads may make up a majority of the τau burden in AD [20]. Both NFT and Aβ plaques must be present in a patient’s brain to earn them a definitive clinical diagnosis of AD, a process that, as of today, can only occur postmortem [21].

#### 1.1.3. Familial and Sporadic Alzheimer’s Disease, and Other Symptoms

Alzheimer’s disease can be broadly classified into two types: familial or early-onset AD (fAD) and sporadic AD (sAD). Familial AD, which accounts for a small percentage of AD cases, arises due to a mutation in the amyloid precursor protein (APP) gene or the PSEN-1 or PSEN-2 genes [22]. APP is a membrane protein whose successive cleavage by β- and γ-secretases produces a membrane-bound C-terminal fragment and Aβ peptides of varying length while cleavage by α- and γ-secretases produces a similar C-terminal fragment and non-amyloidogenic fragment p3 [23]. Mutations in the APP gene alter its α-, β-, and γ-secretase cleavage sites, usually in a way that either increases overall Aβ production or increases the ratio of AB42 to AB40 [24]. PSEN-1 and -2 are part of the γ-secretase complex that sequentially cleaves APP, and mutations in this gene typically increase the ratio of AB42 to AB40 [25,26]. Though fAD accounts for only a small percentage of AD cases, its genetic markers are easily linked to AD pathology. For this reason, fAD mutations are common aspects of AD model systems. 

The implications of fAD’s amyloid-related causal mutations have informed Alzheimer’s research since their discovery. The leading hypothesis of the past three decades, known as the “Amyloid Cascade” hypothesis, suggests that deposition of large quantities of Aβ peptide initiates a “cascade” of other AD-linked pathophysiological symptoms, including the production of tau-containing neurofibrillary tangles [27] and eventual degeneration of neurons [28]. Experimental evidence supports the idea that Aβ acts as an early trigger of AD. For example, mutations in either APP or PSEN genes occur in most fAD cases [29], a finding that strongly implicates Aβ as a potential cause of the disease. Moreover, many studies confirm the link between Aβ deposition and NFT formation [30,31], suggesting that AD tau pathology, at least, may trace its origin back to Aβ. 

Recently, however, the amyloid cascade hypothesis has been subjected to reservations by some scientists as research into sporadic AD, which accounts for the vast majority of AD cases, revealed that factors such as glial cell activation, inflammation, and Aβ clearance play a central role in AD, and that overall Aβ burden does not directly correlate with disease progression, indicating that the amyloid cascade hypothesis alone may not fully capture the complexity of the disease [32,33,34]. Others, however, still cling to the amyloid cascade hypothesis, though increasing numbers of supporters now propose a more holistic mechanism that emphasizes the importance of Aβ clearance and the processes that contribute to it while still upholding the primacy of Aβ in AD pathogenesis [35,36,37]. Presently, the debate over the degree to which Aβ plays a direct role in AD pathogenesis is further impelled by the recent approval of aducanumab, within the scientific community, with some scientists either championing its dismissal while others supporting its primacy [36,38,39].

The most notable genetic marker linked to sAD is the APOE4 allele of the apolipoprotein E (APOE) gene, which codes for a glial lipoprotein involved in, among other things, Aβ trafficking and clearance. APOE4-heterozygous individuals have a three-fold risk of developing sAD compared to APOE3-carrying individuals, and for homozygous individuals, this risk increases almost fifteen-fold. The APOE2 allele, on the other hand, reduces AD risk [40,41]. The discovery that the APOE4 allele confers significant AD risk shifted focus away from the mechanisms of Aβ and p-tau generation and towards the role of Aβ and p-tau clearance in the disease process and compelled researchers to evaluate the importance of the whole CNS microenvironment involved in neuronal Aβ and p-τau generation. 

Naturally, this recent shift to a holistic view of AD also prompted an investigation into its non-proteinaceous pathologies. Though Aβ and p-tau form its primary pathological hallmarks, AD is not solely a proteinopathy and involves a variety of other pathophysiological symptoms. These symptoms include aggregation of activated microglia, dystrophic or p-tau-positive neurites, and reactive astrocytes around plaques as well as granulovacuolar degeneration, cerebral amyloid angiopathy (the deposition of Aβ in cerebral blood vessels), and blood-brain barrier (BBB) dysfunction [42,43] (Figure 1). Brain microcirculation also plays a key role of in AD pathogenesis. AD patients display increased degenerated string capillaries, which are indicative of microvascular dysfunction and loss of functional capillaries and brain volume [44]. Interestingly, apolipoprotein ε4 carriers show higher string vessel counts relative to non-ε4 carriers. Furthermore, cortical cholinergic afferents that contribute to arteriolar vasoregulation as well as markers of noradrenergic vascular innervation are reduced in AD patients, suggesting impaired control of vasodilation and vasoconstriction, respectively, in sAD patients [45]. Many studies suggest that Aβ deposition plays a causative role in p-tau production and NFT pathology [46]. However, the mechanism that links the two pathologies remains elusive, as does the linkage between Aβ deposition, NFT formation, and the neurodegeneration that causes AD’s crippling symptoms. The relationship between these varying pathologies occupies the bulk of current AD research. 

It has become increasingly clear that developing both experimental and theoretical models of AD is crucial to both a better understanding of its pathogenesis and to an assessment of the potential of novel therapeutic approaches. Thus, consideration must be given to developing models in which inflammation [47], prion-like spreading [48], gut-brain interactions [49,50], or pathogenic triggers [51], vasoconstrictions constitute the disease’s main mechanism of pathogenesis. 

## 2. Modeling Alzheimer’s Disease

### 2.1. Computational Modelling 

Within the past decade, computational modeling has become an increasingly powerful tool for the investigation of human diseases, especially proteinopathies like AD. Computational models involving tau and Aβ provide specific, quantifiable information about the aggregatory mechanisms of the two proteins that could prove essential to our understanding of the disease’s pathogenesis. Specifically, molecular dynamics simulations [52] facilitate many such mechanistic studies. For example, Leonard et al. [53] successfully computed the free energy profile for the dissociation of a single tau monomer from the end of an NFT-style protofibril using metadynamics and non-equilibrium steered MD center-of-mass pulling simulations. In so doing, they described a detailed folding pattern by which a tau monomer associates with existing protofibrils (assuming that the dissociation and association processes of tau are reverses of one another). This folding pattern changed for differently shaped protofibrils, suggesting that tau seeding in AD pathogenesis is dependent upon tau protofibril morphology. Detailed mechanisms like this one could disclose specific, aggregation-critical sequences and regions of Aβ and tau for targeted drug design. 

Additionally, molecular dynamics simulations can reveal the mechanisms by which a drug of interest interacts with aggregated Aβ or tau. Wan et al. [54], for example, used molecular dynamics simulation to elucidate the mechanisms by which norepinephrine destabilizes tau protofilaments, and Fan et al. [55] used the same technology to demonstrate a mechanism by which wgx-50 (a natural compound of potential use as an AD-modifying drug) destabilizes Aβ protofibrils. Both studies characterized possible ways in which potentially disease-modifying compounds (wgx-50 and norepinephrine) destabilize the Aβ or p-tau protofibrils that make up amyloid plaques and NFTs. This destabilization would break up amyloid plaques and NFTs and, ideally, lessen AD pathology in patients with the disease. 

Recently, Petrella and co-workers [56] incorporated known clinical biomarkers of AD progression into a computational model to determine the major pathoetiologic manifestations of AD. They simulated biomarker evolution and cognitive decline in early and late-onset disease settings as well as settings mimicking treatment of AD pathology. Biomarkers included Aβ, tau, neuronal loss biomarkers, and cognitive impairment as nonlinear first-order ordinary differential equations (ODEs). These computational models demonstrated the initial appearance of amyloid, followed by biomarkers of tau and neurodegeneration and the onset of cognitive decline as expected based on previous studies. In a separate pioneering computational study, Massimo and co-workers demonstrated that ventral tegmental area (VTA) degeneration may lead to system-level modifications of catecholamine release, which occurs in some AD pathologies [57]. These changes include a midfrontal-driven compensatory hyperactivation of both VTA and norepinephrine, leading to the progression of the VTA loss by a downregulation of catecholamine release and neural degeneration at the cortical and hippocampal levels due to the chronic loss of norepinephrine. Thus, computational modeling can provide vital information about molecular level changes, network dynamics in the initiation and progression of neuropathology in AD [58] and may facilitate the development of improved treatments for AD [59]. However, it is important to appreciate that computational models, though useful, are based on mathematical algorithms. These models can provide new insights, expand understanding, and assimilate information from a variety of standpoints across a range of length scales; however, computational models alone cannot substitute for experiments [60,61], nor can they validate mechanistic aspects that may occur in vitro or in vivo. Thus, computational tools must be merged with experimental disease models to validate computational results and allow researchers to explore a broad variety of potential disease pathways and treatments. 

### 2.2. Experimental Models of Alzheimer’s Disease

#### 2.2.1. Limitations of Transgenic Mouse Models

Initially, AD research relied primarily on studies of tissue from the brains of deceased dementia patients [62]. These tissues offered ample information regarding the disease’s physiological characteristics but provided no ethical way to experimentally investigate the intricacies of the disease processes involved. Newer biomedical research techniques allow researchers to model AD in vitro and in vivo. Such models include 2D cell culture and transgenic mice. Much of what we now know about AD pathogenesis arises from these mouse models of the disease [63]. First-generation mouse models involved transgenic mice that overexpressed APP with or without fAD mutations using various promoters such as prion protein (PrP) and Thy1 [64]. These mice showed extracellular Aβ deposits in the brain, albeit with some variances. However, they were unable to form NFTs or exhibit complete neuronal loss. Thus, the second-generation transgenic mouse models were developed using APP knock-in methodology to form excessive pathogenic Aβ peptides such as Aβ42. This method relied on the development of humanized sequences in mice via the incorporation of fAD mutations into the endogenous mouse APP gene [65]. However, these mice frequently failed to exhibit tauopathy or neurodegeneration. 

The addition of humanized AD-related mutations to mice and the promising results obtained with fAD; led to further path-breaking studies. The prevalence of sAD over fAD prompted the generation of humanized APOE mice [66,67,68]. Such humanized mice recently allowed investigation of early flexibility deficit in E4 versus E3 mice [69] and modulation of early and late-stage neuroinflammation by docosapentaenoic acid [70]. Additionally, loxP-flanked APOE knock-in mice allowed researchers to control tissue-specific expression of the APOE gene and to therefore determine that lack of hepatic APOE has little effect on Aβ deposition. These models are invaluable to the field but still limited in their ability to produce key AD pathologies—for example, the aforementioned study crossed their APOE mice with APP/PS1 transgenic mice in order to quantify the effects of tissue-specific APOE mutation on Aβ deposition [71]. To produce tau pathology, these APOE models are similarly crossed with MAPT or P130S transgenic mice [72]. 

Mouse models of AD, though useful, frequently fail to adequately replicate the disease’s human pathophysiology; even APP/PS1 mouse models that display Aβ plaque deposition frequently fail to produce human-mimetic neurodegeneration [73]. Furthermore, the mutated forms of APP or Presenilin-1 and-2 that allow these models to produce AD-like pathology are associated with fAD, which is much less common than sAD, and the MAPT and P301S mutations that allow them to produce tau are not associated with AD at all. The need for fAD-unrelated mutations to visualize AD-like pathology severely limits the applicability of these systems to studies of AD pathogenesis and its long hypothesized pre-dementia stage [74]. In studies that search for factors that govern Aβ deposition and tau aggregation, animal models shine; in studies that seek causative elements of the disease, they fall flat. Moreover, their inability to replicate the more symptomatic later-stage forms of AD makes them poor vehicles for preclinical therapeutic screening [75] and likely contributes to the extremely high failure rate [76,77] of AD drugs that go to clinical trials. 

Additionally, Aβ vaccination, a therapeutic method that attracted much attention when it debuted in the late 1990s, appeared extremely promising in animal models of AD; vaccination of transgenic mice with Aβ peptide not only reduced Aβ pathology but also prevented memory loss [78]. Unfortunately, not all of this success translated to human patients. In phase III trials, Aβ vaccines nearly eradicated Aβ plaques from the brains of several AD patients but failed to prevent the patients from developing severe dementia, indicating that Aβ plaques themselves do not produce the cognitive symptoms observed in late AD. Notably, these patients retained significant global NFT pathology, a potential reason for their continued cognitive decline [79]. Mouse models, notoriously unable to naturally recreate human tau pathology, were unable to predict this cognitive decline. The need for improved biomimetic in vitro models of AD that recreate the disease holistically, both for investigation of the relationship between various AD-associated pathological events and for efficient screening of potential therapeutics, is clear. Modern microphysiological systems (MPS) present a provocative route by which Alzheimer’s can be more effectively modeled and its pathogenic causes investigated in vitro. 

#### 2.2.2. Microphysiological Modeling

**Figure 2:**
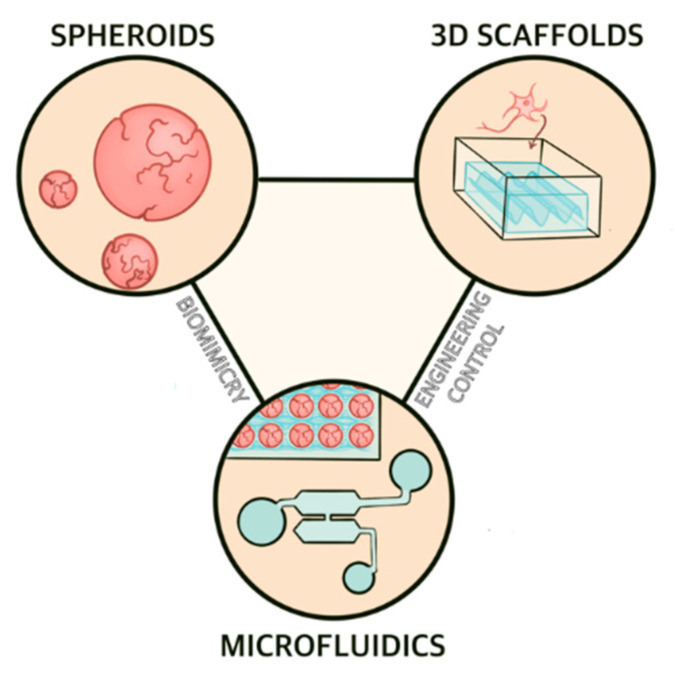
Overview of microphysiological systems for AD modeling. Spheroids offer and 3D scaffold-based cell culture systems provide hanced biomimicry while microfluidic devices combine the two approaches and therefore share both benefits.

Microphysiological systems encompass a host of cell culture platforms whose common purpose is to replicate in vivo physiology in vitro. These technologies include spheroids, organoids, 3D scaffolds, and microfluidic-based chips (Figure 2). A variety of factors make MPS attractive candidates for AD research and modeling. First, MPS for AD applications replicate human neural tissues using human cells (normally iPSC-derived neurons and glia). The use of human tissue and human-mimetic physiology in an AD model avoids some of the issues that make animal models of AD poor predictors of a drug candidate’s efficacy [80]. Furthermore, MPS are typically small, require a (relatively) short timeframe of study and incur (relatively) small costs compared to animal models, making them considerably more attractive for drug screening. Microfluidic devices are especially well-suited for drug screening applications, as many can be designed so that multiple formulations of a drug of interest can be rapidly analyzed on a single chip [81]. In combination with microelectrode arrays, MPS can provide detailed electrophysiological information about culture as a measure of neuronal function [82,83,84], a powerful tool in studies of neurodegeneration. Finally, MPS offer the potential for use in personalized medicine, an attractive approach for treating AD. 

Though a variety of cell lines make up modern MPS, human-induced pluripotent stem cells (hiPSCs) offer compelling advantages to microphysiological models of AD. iPSCs can differentiate into any neural cell type [85] but avoid the ethical concerns associated with fetal and embryonic stem cells. Moreover, AD patient-derived iPSCs allow researchers to directly compare observed in vitro disease processes to those occurring in an actual patient, a powerful way to improve translatability in AD models. Given the as yet undetermined nature of sporadic AD pathogenesis, sAD patient-derived iPSCs are especially interesting for studies investigating its early stages.

Initially, several researchers attempted to create iPSC-based models of AD using two-dimensional cell culture. Though traditional 2D neural cell cultures also offer some of the advantages of three-dimensional models, MPS enjoy significant advantages over traditional 2D neural cell cultures because of their three-dimensional nature. Neural cells have specific morphological and electrophysiological needs that two-dimensional systems cannot meet, causing a vast rift between the behavior of neurons in 2D cultures and neurons in native tissue [86]. 2D models of AD, for example, struggle to produce protein aggregates, even when they successfully manifest elements of AD pathology like Aβ secretion and cannot meet all of the morphological and electrophysiological requirements [87]. 3D culture systems shrink this rift significantly by providing a more relevant 3D microenvironment [88,89] and by facilitating interactions with other CNS cell types that are not present in most 2D cultures. 

One of the first groups to expand in vitro human-cell studies of AD into three dimensions, Choi et al. (2014) [90], did so in an attempt to create an AD model that produced insoluble Aβ plaques. Diffusion of Aβ into the high volume of media required for 2D culture, they suspected, likely prevented the deposition of insoluble Aβ plaques; they hoped that 3D cultures, which are considerably denser, may succeed where 2D cultures failed. To test this concept, they genetically modified commercial human neural progenitor cells (ReN cells) to overexpress fAD-mutated forms of APP and PSEN-1. Consistent with their hypothesis, the team found insoluble extracellular deposits of Aβ and intracellular accumulation of p-tau after a 6-week differentiation. When antibody-labeled and imaged, the tau aggregates closely resembled those of AD patients. Treatment with β- and γ-secretase inhibitors prevented this p-tau accumulation, a result that supports the idea that Aβ acts as an early trigger of AD and that 3D cultures are relevant model systems for AD. Current researchers still use the procedures developed by Choi et al. (2014) to model AD in vitro; For instance, Kwack et al. [91] recently used the hNPC-generation protocol established by Choi et al. to confirm that Aβ42/40 ratio, not overall Aβ level, correlates with tau pathology in 3D cell culture models of AD.

**Figure 3:**
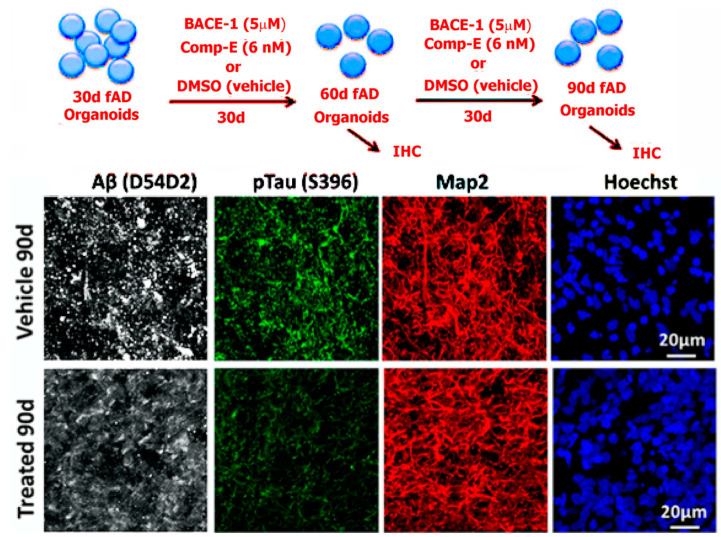
**Top**: Scheme showing fAD organoids grown for 30 days followed by treatment with beta-secretase and gamma-secretase (comp E) inhibitors at varying concentrations. **Bottom**: left to right shows organoids derived from multiple familial AD (fAD) patients with APP duplication or PSEN1 mutation, compared to controls at 90 days of culture. The organoids were processed for immunohistochemistry. Tissue sections from fAD and control organoids were processed for immunoreactivity against Aβ (D45D2, white), pTau (Ser396, green), and MAP2 (red). It was shown that treatment of patient-derived organoids with β- and γ-secretase inhibitors significantly reduced amyloid and tau pathology. Reproduced from reference [59], Raja et al. (2016). PLoS ONE 11(9): e0161969. (This is an open-access article distributed under the terms of the Creative Commons Attribution License, which permits unrestricted use, distribution, and reproduction in any medium, provided the original author and source are credited).

Choi et al.’s approach, though elegant, suffered insofar as it required overexpression of fAD-linked mutations—such overexpression is not reminiscent of in vivo AD pathogenesis, and an ideal model would develop AD pathology naturally. Thus, Raja et al. [92] later produced a scaffold-free 3D culture model of AD using fAD patient-derived iPSCs (Figure 3); this model developed Aβ deposition and NFT pathology after 100 days of culture without overexpressing fAD-linked mutations. These two foundational papers, both of which applied 3D tissue culture approaches to AD modeling, provided the framework for many current 3D culture-based models of AD. 

#### 2.2.3. Three Dimensional Scaffolds

3D scaffold-based models of AD have long captured the attention of researchers, as they allow optimization of size, shape, porosity, and tensile strength of their matrices. Scaffold materials are usually selected to fit the paradigms of a particular question of interest. The most used scaffold for 3D neural cell cultures is Matrigel, but others have opted for more specialized tissue-engineering materials, including alginate-gellan gum-laminin blends [93], microfibrous PLGA [94], designer self-assembled peptides [95], and decellularized porcine brain ECM [96], which has also been tested as an external scaffold for spheroids [97]. Likewise, an early adopter of 3D culture for AD modeling, Zhang et al. [98], created iPSC-derived neuronal organoids in a 3D matrix made of RADA-16, a self-assembling amphiphilic peptide. RADA-16 offers numerous advantages to tissue culture; it is biocompatible, highly stable, commercially available, and is able to self-assemble into amyloid-like nanofibrils around dissociated cells, promoting a homogeneous distribution pattern [99]. After generation, Zhang et al. treated the organoids with exogenous Aβ and measured differences in expression of P-21 activated kinases (PAKs), mechanotransduction-involved proteins whose levels diminish in late-stage AD, between 2D and 3D cultures. The 3D cultured cells displayed lower levels of PAK where the 2D culture did not, showing that 3D scaffolds offer a more physiologically relevant microenvironment for cell culture models of AD compared to 2D cultures. Unfortunately, Zhang et al.’s model is not ideal for most current AD-modelling applications as RADA-16 becomes disrupted after multiple media changes, making it unsuitable for protocols requiring more than two weeks of culture time. Moreover, the team actively avoided using glial cells in their model by differentiating the iPSCs through a neuroepithelial pathway. The current opinion considers glial cells vital to AD processes [100,101,102], making majority-neuronal AD models like the one used in this study less than helpful. Even so, Zhang et al.’s successful use of RADA-16 suggests a role for creative use of scaffold materials in AD modeling and provides an excellent base for an AD model that requires little culture time. 

Another group, Papadimitriou et al. [103], used a star-shaped polyethylene glycol (starPEG)-Heparin scaffold for their model of neural stem cell plasticity loss in AD. This material allowed them to optimize neural network formation and astrocyte neurogenic plasticity to a greater degree than Matrigel. To determine how Aβ42 impairs neural stem cell plasticity and whether restoring NSC neurogenic activity would alleviate AD symptoms, they treated their system with exogenous Aβ and observed that, as expected, Aβ treatment results in toxicity and impaired neurogenesis. Notably, treatment with interleukin-4 reduced the toxic effects of Aβ in these cultures, a potentially important observation in the pursuit of a regenerative neural stem cell-based treatment.

Unfortunately, many of these 3D models rely on overexpression of familial AD-causing mutations or treatment with exogenous Aβ; though these methods effectively achieve the AD phenotype in vitro, they do not accurately replicate the hyper-prevalent sporadic form of the disease’s pathogenesis, which remains mercurial. Thus, more recent work has focused on the development of an AD model that produces an AD phenotype in a way that reflects current (if tentative) hypotheses regarding the disease’s pathogenesis. 

**Figure 4:**
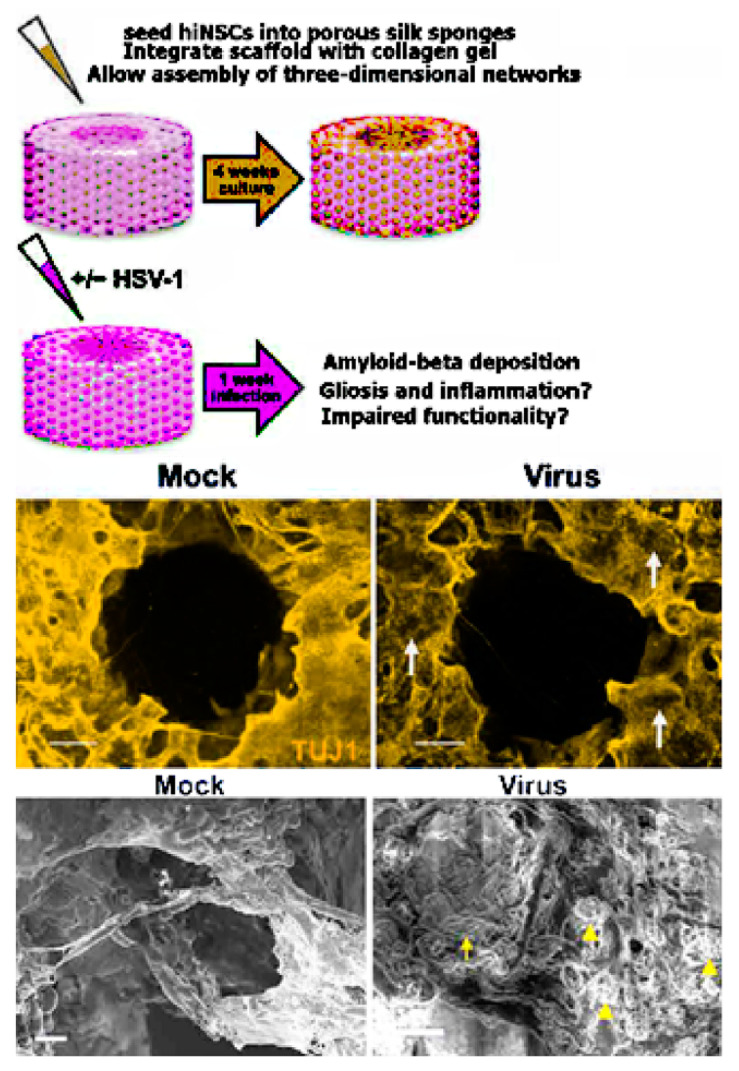
Human-induced neural stem cells (hiNSCs) cultured in a 3D brain model developed an AD-type phenotype in response to low-level HSV-1 infection. For mock infections, an equal volume of control culture medium from uninfected Vero cells was used. (**Top**) Model of 3D human brain–like model. hiNSCs were cultured in the donut model for 4 weeks before HSV-1 infection for 1 week. (Middle) Images showing β-III tubulin) and Aβ immunostaining. Arrows point to regions of neuronal loss in HSV-1–infected tissues. (**Bottom**) SEM images showing changes in HSV-1–infected tissue constructs. Arrowheads and arrow indicate the presence of both small and relatively larger plaque link formations, respectively. Scale bars, 10 µm. Reproduced from reference [85], Cairns et al., Sci. Adv. 2020; **6**: eaay8828 6 May 2020. (This is an open-access article distributed under the terms of the Creative Commons Attribution License, which permits unrestricted use, distribution, and reproduction in any medium, provided the original author and source are credited).

One such novel hypothesis-based 3D scaffold AD model was pioneered by Cairns et al. [104]. The group developed a 3D brain tissue model system precisely engineered to mimic the structure of native brain tissue, including regions of white and grey matter. This “top-down” system used a porous silk protein “donut” infused with type I collagen gel and seeded with wild-type human induced neural stem cells (hiNSCs), allowing for measurement of electrical activity and visualization of morphological properties as shown in Figure 4. The soma of the neural cells remained within the silk protein, mimicking grey matter, while the type I collagen promoted neurite outgrowth into the center of the donut, mimicking white matter. Notably, hiNSC generation bypasses the pluripotent state, a potentially important step in the study of age-related diseases. Cairns et al. infected their model brain donut with a low level of herpes simplex virus 1 (HSV-1). The infected model brains formed large, Aβ and p-tau-positive multicellular plaque-like formations like those found in AD brains. Electrophysiological activity decreased in these cultures, suggesting a loss of network functionality. The infected cultures also displayed signs of reactive astrocytes and neuroinflammation, aspects of the disease that other culture systems have been unable to reproduce. Treatment with an antiviral decreased the severity of these phenotypes dramatically. This model system is intriguing not only for its replication of multiple AD phenotypes, but also for its short experimental timeframe and ease of replication--the cultures developed AD pathology less than one week after infection whereas both patient-derived fAD and sAD models and many fAD mutation-overexpressing systems require months to produce the same effect. Moreover, this system is unique in 3D scaffold-based AD models as it mimics multiple brain regions, including separated regions of grey and white matter, by organizing multiple scaffold materials in a controlled manner.

In any scaffold-based model, thought must be given to the way in which the properties of the chosen scaffold affect Aβ aggregation and Aβ-mediated cytotoxicity. Simpson et al. [105] recently investigated the effect of hydrogel mesh size on Aβ-mediated cytotoxicity and Aβ aggregate size and structure. They found that 3D scaffolds alleviate some Aβ-related cytotoxicity compared to 2D cultures, a phenomenon that is not related to mesh size or bioactivity, but instead likely results from the rapid stabilization of larger, less toxic Aβ aggregates that is common to 3D hydrogels. All the hydrogels studied, however, affected Aβ aggregation kinetics, demonstrating the importance of understanding the way artificial microenvironments affect Aβ aggregation in AD models. For instance, laminin, an essential component of Matrigel, has a high affinity for Aβ and inhibits Aβ fibril formation [106]. This property must be considered when evaluating Matrigel-based AD models.

Although neural activities in 3D in vitro models are most commonly monitored using calcium imaging or electrophysiology with 2D microelectrode arrays, analyzing the neuronal networks and circuitry in a 3D microenvironment [107] or in vivo is relatively difficult using these methods alone [108]. Recently, Shin and co-workers created a novel three-dimensional high-density multifunctional microelectrode (HDMFA) array capable of optical stimulation and drug delivery for probing neural circuit dynamics within engineered 3D neural tissues [109]. This array provided precise measurements of synaptic latencies in 3D neural networks. They implemented the 3D HDMFA using a stacking method [110] that allowed for exact mapping of functional connectivity between neurons in the complete 3D neural tissue. Such innovative microelectrode arrays may open up new avenues for investigating neural circuit dynamics within 3D brain models as well as neural circuit degeneration in 3D AD models. 

#### 2.2.4. Spheroids

Compared to 3D scaffold-based models, spheroids offer relatively less engineering control, but significantly more biomimetic character. Spheroids and organoids refer to stem cell-derived 3D tissue culture systems that mimic the structure of in vivo tissue [111]. As some of the simplest organoids, self-organizing spheroids require little engineering. These spheroids develop naturally from stem cell lines if given a suitable culture environment such as differentiation-inducing media and a low-attachment culture dish. Other organoids, however, involve extensive tissue engineering. These “scaffold organoids” typically involve the use of scaffold material and directed patterning, usually intended to better mimic the natural patterning of the cells in the brain and to aid the diffusion of culture media throughout the cell microenvironment. 

Spheroids are one of the most popular current organoid AD modeling systems, likely due to their self-assembling abilities and their relatively longer pedigree of generation protocols compared to other stem cell-based culture systems. Indeed, another research group produced an updated version of Raja et al.’s fAD patient-organoid generation protocol using more modern organoid-generation techniques as recently as 2021 [112]. Specifically, this group used an embedded-Matrigel generation protocol and created an isogenic control patient-derived iPSC population without the patient’s native PSEN-1 mutation using CRISPR/Cas9 technology. 

Such patient-derived spheroids have already begun to make their debut as tools for studying specific molecular aspects of the disease’s pathogenesis. Arber et al. [113], for example, used iPSC-based spheroid cultures to examine the effects of different fAD mutations on the production levels of a spectrum of Aβ peptides, including AB38, AB40, AB42, and AB43. The team cultured iPSCs from seven fAD patients for extended periods of time (100–200 days) alongside non-AD controls in both 2D and 3D cultures, allowing Aβ secretion to occur endogenously. The overall levels of Aβ produced by these cultures varied greatly, even in iPSC-derived cultures from the same patient. However, the ratios between Aβ variants (Aβ 38, Aβ 40, Aβ 42, and Aβ 43) were consistent between cultures from the same source. The team found that different fAD-causing mutations altered these ratios significantly, allowing them to propose that different mutations affect different Aβ-generating pathways, thus altering the AB42:40 ratio.

Though fAD-based spheroids are useful for proof-of-concept and preliminary studies, the eventual goal of such technologies is to provide insight into sAD pathogenesis and treatment. Thus, following the success of fAD-based spheroids, many researchers used similar techniques to create sAD-based spheroids. One such group, Lee et al. [114], produced five patient-derived sAD neurospheroids in vitro. Though four of the five patient-derived spheroids showed significantly reduced Aβ burden upon treatment with BACE1 inhibitor, one spheroid showed little response to the treatment. Further analysis showed that this cell line had higher levels of BACE1 substrate APP compared to the other four, indicating that genotypic variation might be responsible for its resistance to the BACE1 inhibitor. This result suggests that individual sAD patients may harbor differences in APP expression that affect their dose-dependent response to amyloid clearing drugs. Such variation, so prominent in these patient-derived cultures, would be nearly impossible to predict using animal models of AD. 

More recently, Lin et al. [115] examined the difference between isogenic APOE4 and APOE3 iPSCs in organoids. They found that, as expected, APOE4 iPSC-derived organoids displayed AD phenotypes, though these phenotypes occurred at six months as opposed to two, as in Raja et al.’s fAD organoids. Moreover, neurons, astrocytes, and microglia-like cells displayed different disease phenotypes, in accordance with the cellular hypothesis of AD pathogenesis. Specifically, the neurons showed an increase in synapse number, endosomal abnormalities, and Aβ42 secretion, whereas astrocytes and microglia-like cells showed impaired Aβ uptake. Cell type-specific studies like this one allow for the determination of type-specific responses to hypothesized AD triggers and are therefore useful to any effort to elucidate the disease’s pathogenesis. 

Despite the advances made in their application as AD modeling systems, spheroids retain several endemic flaws. Unfortunately, spheroids suffer from a lack of vascularization; culture media and any drugs of interest within it cannot efficiently penetrate the interior layers of the spheroid. The maximum size and age of spheroids are therefore severely limited, hindering their ability to model age-related diseases [116]. Traditional spheroid generation protocols also tend to produce ample heterogeneity in spheroid size, limiting their use for drug screening applications, and most fail to naturally create significant microglial cell populations [117], requiring co-culture with induced microglia-like cells for a multi-cell type model. Overall, vascularization poses the most significant challenge to spheroid models of AD. 

Fortunately, researchers have already begun to tackle the vascularization issue. In their recent work, Rothenbucher and co-workers developed flat brain organoids (fBOs) by growing the organoids with cells seeded in polycaprolactone scaffolds and altering their shape systematically into flat structures [118]. These flat brain organoids demonstrated excellent diffusion conditions, allowing for better oxygen and nutrient supply and hindering the formation of necrotic tissue. Using this method, the organoid size could be tailored; moreover, self-generated folding suggestive of gyrification appeared within three weeks of growth. Such fBOs may be a stepping stone for the generation of stable brain models with enhanced vascularization properties for a variety of studies including spatial patterning, drug screening, and AD modeling. 

Ao and co-workers [119] recently developed a tubular organoid on-a-chip device to generate organoids and model neuroinflammation. To generate the tubular organoids, the researchers designed 3D printed tubular hollow mesh scaffolds with perfusable gaps, which they incorporated into multiwell plates. They then integrated isogenic microglia into the tubular organoid to mimic microglial responses and create a microenvironment similar to that of the native brain. The group also introduced rocking flows through the tubular device channels to encourage nutrient and oxygen flow and reduce necrosis and hypoxia and incorporated immune cells into organoids to model neuro-immune interactions. The tubular organoids showed higher neural progenitor proliferation and reduced heterogeneity of human brain organoids. A similar organoid on a chip device can be devised to mimic neuroinflammation in AD models. 

#### 2.2.5. Microfluidics

**Figure 5:**
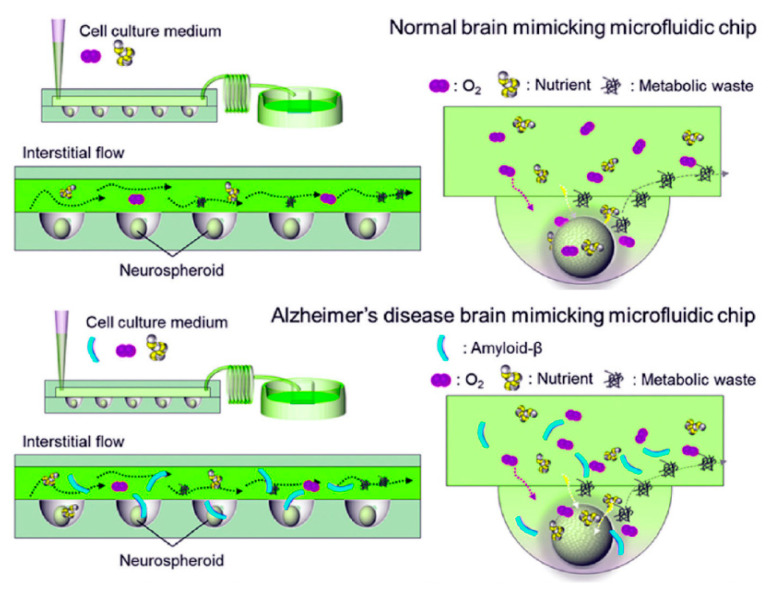
Scheme showing a comparison of (**top**) normal brain mimicking and (**bottom**) AD brain mimicking microfluidic chip. Neurospheroids were cultured under dynamic conditions with a flow of medium containing oxygen and nutrients on each chip for ten and seven days, respectively. In the case of the neurospheroids cultured on AD brain mimicking chip, it was then incubated with a medium containing synthetic amyloid-β (1–42) for an additional three days. (Adapted from Reference [87]. Park et al. (2014). Lab Chip. 2015, 15, 141. Reproduced with permission from Royal Soc. Chem).

In response to the heterogeneity intrinsic to traditional spheroid models of AD, researchers turned to microfluidic technology to provide engineering control over the size and composition of these spherical organoids. In the context of AD research, microfluidic devices typically take the form of “organs-on-chips”, cell culture devices that enhance physiological relevance through the application of mechanical cues such as fluid shear stress, tension, and compression that are normally present in vivo [120]. Some of the advantages of microfluidic technology for use in AD studies, including its use in AD biomarker detection and diagnosis, have been reviewed elsewhere [121]. Initial microfluidic-based cellular models of AD used Aβ concentration gradients to study Aβ toxicity through the incorporation of an osmotic pump-based microfluidic platform with an interstitial level of flow intended to mimic the neural microenvironment [122]. Though this platform used a two-dimensional culture, it became the blueprint from which later studies derived their platforms. Thus, a year later, the same group combined their physiological flow system with three-dimensional neurospheroid cultures and found that the flow-cultured neurospheroids displayed increased size and neural network formation compared to static neurospheroids [123]. To accomplish this, they developed an in vivo-mimicking microfluidic 3D brain-on-a-chip with the interstitial flow by combining concave microwell arrays with an osmotic micropump system to investigate the effect of flow on 3D micro-spheroidal neural tissue (neurospheroids). (Figure 5) This system used both static (without flow) and dynamic (with flow) neurospheroid cultures. Neurospheroids were cultured in parallel with and without amyloid-β, allowing the researchers to mimic normal and Alzheimer’s disease brains simultaneously on a single platform. This type of 3D brain-on-a-chip provided an interstitial level of flow that mimics the in vivo microenvironment and enables long-term monitoring and may aid in further elucidation of pathways in AD [123].

**Figure 6:**
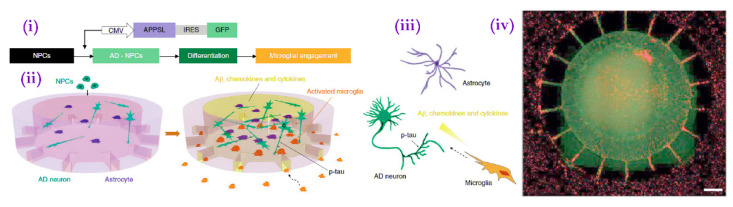
(**i**) Scheme for growth of human AD triculture system (Neuron + Astrocyte + Microglia AD) using a microfluidic platform differentiated from human neural progenitor cells and human adult microglia. CMV, human cytomegalovirus. Scheme showing multicellular 3D layouts in (**ii**) a microfluidic human AD culture model and (**iii**) human AD brain tissue. (**iv**) Fluorescent microphotograph show the layout of human AD neurons/astrocytes (green) in a central chamber and microglia (red) in angular chambers. Scale bar, 250 μm. (Adapted from Reference [81]. Park et al. (2016) Nat. Neurosci. 2018, 21, 941. Reproduced with permission from Springer Nature).

In another study that would become a blueprint for later methods, researchers developed a multi-chambered platform in which microglial migration towards a central compartment containing bound Aβ could be visualized in real time [124]. Though limited by its two-dimensional nature, this platform allowed for the visualization of Aβ-induced microglial chemotaxis. In a more recent study, Park et al. [125] developed a similar multi-chambered microfluidic platform by which they could visualize the migration of activated microglia from peripheral chambers into the central chamber (Figure 6). Unlike its predecessor, however, its central chamber contained hNPC-generated neurons and astrocytes overexpressing fAD-mutated APP created using Choi et al.’s protocol rather than an exogeneous Aβ deposit. Once recruited, the microglia killed the neurons and astrocytes via a cytokine-mediated mechanism. The team then replicated the experiment using fAD mutation-overexpressing iPSCs, with similar results. Studies of similar construction could allow focused investigation of the mechanisms of microglia recruitment and microglia-induced neurotoxicity in AD, especially if combined with more disease-reminiscent pathogenesis models such as APOE4 expression. 

In addition to co-culturing abilities, as described in Park et al., multi-chambered microfluidic devices offer the ability to isolate structures of interest while maintaining a physiologically relevant cell culture. Kilinic et al. [126] took advantage of this ability by isolating the synapses of rat primary neurons and selectively exposing them to physiological concentrations of amyloid beta, thus examining the effects of Aβ on synaptotoxicity. Such multi-chambered devices also provide a platform by which to separate pre- and postsynaptic neurons, a feature that researchers have used to quantitatively study the axonal transfer of tau in rodent cortical neurons [127]. These systems allow focused study not only of different cell types but also of different aspects of neural cells and synapses. 

Recently, Shin et al, [128] modeled AD-induced blood–brain barrier (BBB) dysfunction in a three-dimensional multi-chambered microfluidic chip using fAD mutation-overexpressing ReN (hNPC) cells. This chip included two main chambers, one containing ReN cell differentiation media and the ReN cells themselves, and the other containing type I collagen and a brain endothelial cell (bEC) monolayer. The two chambers were separated by a single microchannel initially filled with air to which the researchers added collagen after differentiation and maturation of the cells in the separate chambers. After the addition of the collagen bridge, the AD chips displayed increased BBB permeability, decreased expression of tight junction proteins, increased ROS generation, and Aβ deposition on the brain endothelial cell barrier compared to wild-type chips. Much like Cairns et al., this model system is exciting for both its cutting-edge biomimetic character and its extremely short experimental timeframe. Ideally, this model could facilitate BBB dysfunction-ameliorating drug discovery, though its relevance as an AD model may be limited due to its use of fAD-overexpressing cells. Additionally, the size of the bEC microchannels used in this model greatly exceeded that of brain capillaries, which make up a majority of the cerebral microvasculature and typically have diameters less than 10 µm. Moreover, AD-related microcirculatory dysfunction is difficult to study in vitro [129], making microfluidic devices an ideal platform by which to investigate this facet of the disease. This microchannel size limitation will therefore need to be addressed if complete biomimicry is to be achieved.

In a separate study, Jorfi et al. [130] developed a protocol for producing large-scale, uniform, microfluidic neurospheroid arrays adapted to 96-well plates, an ideal setup for high-throughput drug screening. Following their success and the success of Park et al. in modeling microglia-mediated neuroinflammation, Cai et al. [131] produced an acoustofluidic spheroid model of neuroinflammation in AD. This model system provided precise control over spheroid size as well as cell type and number and required little time to produce an array of uniform spheroids. Unfortunately, this model required the introduction of exogenous Aβ to the assembly mixture; however, it successfully showcased Aβ-induced microglial activation and migration reminiscent of the AD phenotype.

Cho and co-workers recently demonstrated another robust bioengineering platform to enhance iPSC-derived human cerebral organoid culture integrated with microfluidics. The team built a 3D brain-mimetic microenvironment by combining decellularized human brain tissue-derived brain extracellular matrix (BEM) with dynamic microfluidic systems [132]. The microfluidic system mimicked fluid flow akin to cerebrospinal fluid and provided interstitial spaces that facilitated oxygen and nutrient flow, leading to increased cell viability within the entire organoid. The use of the BEM supported cell expansion and neuronal differentiation and maturation, thereby mimicking key properties of human embryonic cortical development. Furthermore, this microfluidic platform allowed for greater control of the cerebral organoids in much smaller medium volume and controlled medium flow with low fluid shear stress, compared to bulk systems, which require larger volumes and cause cell damage due to high shear stress. Such combined BEM with microfluidics has not yet been used for AD modeling and may represent a useful method to increase biomimicry in these cultures. 

Though a complete biomimetic microfluidic AD brain model is yet to be developed, the devices appear to have a promising future in AD research. The greatest advantage of microfluidic models of AD is their scalability. Most devices allow the researcher to conduct large numbers of experiments simultaneously in the footprint of a single 96-well plate. This scalability is tremendously valuable to drug screening applications, as single experimenters can test large numbers of potential therapeutics simultaneously. Moreover, microfluidic devices excel in promoting neuronal network formation, a vital component in models of a disease that potentially involves the synaptic transmission of disease proteins.

## 3. Conclusions and Future Directions

Despite their many advantages and exciting potential, MPS retain their share of obstacles. Most notable is the expense associated with human cell culture, and the limited availability and difficult reprogramming protocols of patient-derived iPSCs also pose significant problems. Moreover, research teams run into issues with extended cell viability, and any lengthy cell culture protocol requires enormous care and skill to execute without contaminating or otherwise destroying the culture. The need for trained researchers in the field, therefore, limits the applicability of many of these models. Additionally, even leading neural MPS do not wholly mimic the complex structure of the brain. As innovative as current AD-MPS are in promoting network connectivity, they still lack anything resembling the coordinated circuit function present in brain tissues. Finally, even successfully generated iPSC-derived organoids acquire a gene expression profile that closely resembles that of fetal tissue [133], a potentially enormous issue in the study of age-related diseases. To remedy this issue, some researchers have suggested methods by which to bypass the pluripotent state. Whether such protocols benefit AD models remains to be seen. 

Further investigation of iPSCs, specifically, whether age and disease progression of the patient source affect an iPSC-derived culture’s ability to manifest AD pathology, may aid efforts to model neurodegenerative diseases using their cellular descendants. Although children carrying fAD mutations do not apparently develop AD pathology or symptoms, fAD-carrying organoids with fetal gene expression profiles display significant amyloid and τau deposition. The reasons for this discrepancy are worthy of investigation. Other cell types and differentiation protocols should also be investigated for use with such models. Because it is an age-related disorder, neural cell lines generated via direct differentiation protocols that bypass the pluripotent state may be of particular use for AD.

Combining the approaches of extant models could also prove beneficial to future research into AD pathogenesis. For example, testing the effects of viral infection on these models would both test cross-platform reproducibility and provide evidence for or against the use of such protocols alongside or in place of fAD-mutation overexpressing in vitro AD models. An important goal of these model systems is to provide a platform for testing of sAD-targeted therapeutics; however, because sAD pathogenesis is still poorly understood, in this still-early stage of model system development fAD mutation-carrying models predominate. Despite the debatable nature of the viral hypothesis of AD pathogenesis, the appearance of Alzheimer’s-like pathology and especially the speed with which that pathology appeared makes this protocol intriguing. Although sAD patient-derived cells are ideal for studies of sAD pathogenesis, these cells must be cultured for several months to produce significant AD-like pathology. Current models using APOE4-expressing cells require similarly extended culture periods, though the creation of an APOE4 transfectant is considerably more facile than the acquisition of patient-derived cells, making such models attractive for sAD-specific studies. 

Further exchange of cross-model ideas could further optimize microphysiological models of AD. For example, protocols carried out using fAD cells should be repeated with sAD patient-derived cells and/or APOE4-expressing cells, especially if the models in question have already demonstrated an ability to survive long culture periods. Additionally, multi-organ chips designed to mimic the gut-brain axis and other key multi-organ interactions of potential importance in AD pathogenesis represent an important next step for in vitro AD models.

Spheroids, 3D scaffold cultures, and microfluidic cultures each offer unique advantages as cellular models of AD (Table 1). Spheroids replicate the organization of physiological brain tissue, but do not provide engineering control over the size and composition of the culture and therefore carry issues with replicability. 3D scaffolds provide more control over the cellular microenvironment, but in many cases, this control comes at the expense of physiological relevance. Microfluidic devices that combine the benefits of spheroid cultures and 3D scaffolds may be critical for developing the next generation of AD models. These provide engineering control over spheroid size, composition, and microenvironment and allowing the separation of different cell types into multi-chambered co-cultures, and administer physiological levels of fluid flow that enhance the translational salience already present in these human cell culture models of AD.

Regardless of their present limitations, optimized MPS could prove revolutionary to the field of AD research. Their translatability will be particularly useful to drug design, especially if combined with the large-scale microfluidic arrays that allow testing of hundreds of compounds at once. Addressing the present challenges faced by MPS is therefore essential to Alzheimer’s research as a whole.

## Figures and Tables

**Table 1:** Pioneering Models Studied in Alzheimer’s Disease.

Study	Technology Used	Cell Type and Treatment	Results	Year
Choi et al.	Matrigel as 3D scaffold	Commercial hNPCs overexpressing FAD-mutated APP and PSEN-1	Creation of fAD-mutated hNPC line with the following characteristics: Increased overall AB levels at 6 weeks, inc. AB 42:40 ratio in some mutated cell lines. Both AB and tau pathology, including insoluble extracellular AB deposits and intracellular tau aggregates. Inhibition of AB production → dec. tau pathology GSK-3 regulated AB-mediated tau phosphorylation	2014
Raja et al.	Spheroid	fAD patient-derived iPSCs (multiple cell lines)	Creation of AD patient-derived cerebral organoid with the following characteristics: Spontaneous AB accumulation and aggregation, subsequent spontaneous p-tau accumulation Endosome abnormalities. Treatment with B- and y-secretase inhibitors subsequently reduced AB deposition, then p-tau accumulation	2016
Lee et. al.	Spheroid (see Raja et al.)	sAD patient-derived iPSCs (multiple cell lines)	BACE1 and y-secretase inhibitors reduced AB levels in some, but not all, patient-derived cell lines Reduced efficacy of inhibitors in spheroids compared to 2D culture, likely a result of decreased diffusion	2016
Lin et al.	Spheroid co-culture	Isogenic AOPE4/APOE3 iPSCs differentiated into neurons, astrocytes & microglia. APOE4 iPSCs were generated by editing APOE3 iPSCs using CRISPR/Cas9 gene editing.	APOE4 organoids displayed heightened AD phenotypes compared to APOE3 organoids at 6 months	2018
Park et al.	Multi-chambered microfluidic triculture system	Commercial hNPCs overexpressing fAD-mutated APP and differentiated to neurons and astrocytes (see Choi et al.). Repeated with commercial iPSCs.	fAD neurons and astrocytes in the central chamber induced activation and migration of microglia added to the peripheral chambers toward the central chamber and mimicked AD pathologies including AB aggregation, p-tau accumulation, and neuroinflammation.	2018
Shin et al.	5-chambered PDMS Microfluidic BBB-on-a-chip	Commercial hNPCs overexpressing fAD-mutated APP and APP/PSEN1 (see Choi et al.) Commercial brain endothelial cells	Increased bEC monolayer permeability, decreased expression of tight junction proteins, and vascular endothelial AB deposition upon co-culture with fAD-expressing cells	2019
Cairns et al.	Engineered multi-sectional 3D scaffold infected with HSV-1	Human-induced neural stem cells generated from foreskin fibroblasts through direct reprogramming (bypasses the pluripotent state)	Generation of AB and p-tau positive plaques, reactive astrocytes, and neuroinflammation, as well as loss of network functionality	2020
